# Prediction of Other-Cause Mortality in Older Patients with Breast Cancer Using Comorbidity

**DOI:** 10.3390/cancers13071627

**Published:** 2021-04-01

**Authors:** Anna Z. de Boer, Esther Bastiaannet, Hein Putter, Perla J. Marang-van de Mheen, Sabine Siesling, Linda de Munck, Kelly M. de Ligt, Johanneke E. A. Portielje, Gerrit Jan Liefers, Nienke A. de Glas

**Affiliations:** 1 Department of Surgery, Leiden University Medical Center, 2333 ZA Leiden, The Netherlands; a.z.de_boer@lumc.nl (A.Z.d.B.); e.bastiaannet@lumc.nl (E.B.); g.j.liefers@lumc.nl (G.J.L.); 2 Department of Medical Oncology, Leiden University Medical Center, 2333 ZA Leiden, The Netherlands; j.e.a.portielje@lumc.nl; 3 Department of Medical Statistics, Leiden University Medical Center, 2333 ZA Leiden, The Netherlands; h.putter@lumc.nl; 4 Department of Medical Decision Making, Leiden University Medical Center, 2333 ZA Leiden, The Netherlands; p.j.marang-van_de_mheen@lumc.nl; 5 Department of Research and Development, Netherlands Comprehensive Cancer Organization, 3500 BN Utrecht, The Netherlands; s.siesling@iknl.nl (S.S.); l.demunck@iknl.nl (L.d.M.); k.d.ligt@nki.nl (K.M.d.L.); 6 Department of Health Technology and Services Research, Technical Medical Center, University of Twente, 7522 NB Enschede, The Netherlands

**Keywords:** breast cancer, comorbidity, competing for mortality, older patients, individualized treatment

## Abstract

**Simple Summary:**

Selecting older patients for adjuvant breast cancer treatments is challenging as its benefits can be diminished by shorter life expectancies. In addition to age, comorbidity increases the risk of dying from other causes than breast cancer. Available prediction tools have either not adjusted for individual comorbidities or have shown inaccurate predictions when a higher number of comorbidities are present. Up to now, an optimal comorbidity score to be used in prediction tools has not been established. Therefore, this study aimed to assess the predictive value of the Charlson comorbidity index for other-cause mortality and to compare these predictions with using a simple comorbidity count. We found that the Charlson index performed similarly as comorbidity count. The use of comorbidity count in the development of new prediction tools for older patients with breast cancer is recommended as its simplicity enhances the tool’s applicability in clinical practice.

**Abstract:**

*Background*: Individualized treatment in older patients with breast cancer can be improved by including comorbidity and other-cause mortality in prediction tools, as the other-cause mortality risk strongly increases with age. However, no optimal comorbidity score is established for this purpose. Therefore, this study aimed to compare the predictive value of the Charlson comorbidity index for other-cause mortality with the use of a simple comorbidity count and to assess the impact of frequently occurring comorbidities. *Methods*: Surgically treated patients with stages I-III breast cancer aged ≥70 years diagnosed between 2003 and 2009 were selected from the Netherlands Cancer Registry. Competing risk analysis was performed to associate 5-year other-cause mortality with the Charlson index, comorbidity count, and specific comorbidities. Discrimination and calibration were assessed. *Results*: Overall, 7511 patients were included. Twenty-nine percent had no comorbidities, and 59% had a Charlson score of 0. After five years, in 1974, patients had died (26%), of which 1450 patients without a distant recurrence (19%). Besides comorbidities included in the Charlson index, the psychiatric disease was strongly associated with other-cause mortality (sHR 2.44 (95%-CI 1.70–3.50)). The c-statistics of the Charlson index and comorbidity count were similar (0.65 (95%-CI 0.64–0.65) and 0.64 (95%-CI 0.64–0.65)). *Conclusions*: The predictive value of the Charlson index for 5-year other-cause mortality was similar to using comorbidity count. As it is easier to use in clinical practice, our findings indicate that comorbidity count can aid in improving individualizing treatment in older patients with breast cancer. Future studies should elicit whether geriatric parameters could improve prediction.

## 1. Introduction

Over 30% of patients diagnosed with breast cancer are 70 years or older [1]. The risk of dying from other causes than breast cancer strongly increases with age [2,3]. Nine years after diagnosis, 21% of the patients aged 70–74 years have died from other causes compared to 61% over 80 years [3]. Selecting patients for adjuvant treatments is one of the challenges for clinicians who are treating this patient population since the effect of radiotherapy, endocrine therapy, or chemotherapy can be diminished by shorter life expectancies. The benefit of adjuvant treatments in patients that are likely to die from other causes is, therefore, questionable [4,5,6]. Hence, it is essential to take this other-cause mortality into account when estimating prognosis and treatment benefit [2,3]. In addition to age, the presence of comorbidity is an important determinant for other-cause mortality [7,8].

The PREDICT tool has been demonstrated to accurately predict overall survival in older patients with breast cancer, but its implications for treatment decisions are unclear as mortality from breast cancer and other causes are not adjusted for individual comorbidities. Indeed, the predictions are less accurate if patients have multiple comorbidities [9]. The currently unavailable Adjuvant! Online tools did predict both cancer-specific and other-cause mortality, but inaccurate predictions were reported in patients over 65 years, especially when a higher number of comorbidities were present [10]. One proposed explanation is that Adjuvant! Online does not define the incorporated comorbidity categories (including, for example, “minor problems” or “average for age”).

Up to now, an optimal comorbidity score to be used in prediction tools that aid in individualizing treatment decisions in older patients with breast cancer has not been established. The Charlson comorbidity index is frequently used to describe study populations’ general health status and adjust for differences in comparative effectiveness studies [11,12]. The Charlson index comprises sixteen comorbidities, of which three are assigned extra weight. Since the Charlson index is widely known, it could be convenient to use it as a comorbidity score in a prediction tool. On the other hand, relevant comorbidities that are not included may be missed, and calculating the Charlson score requires some extra time from the clinician.

Therefore, the aim of this study was to assess the predictive value of the Charlson comorbidity index for other-cause mortality and to compare these predictions with using a simple comorbidity count. In addition, the aim was to assess the impact of frequently occurring comorbidities on 5-year other-cause mortality.

## 2. Methods

### 2.1. Design and Patients

This study was a nationwide population-based cohort study. Patients were selected from the database of the Netherlands Cancer Registry (NCR), of which data are currently used in over 200 publications annually (https://iknl.nl/en/ncr, accessed on 1 March 2021). The NCR receives reports of diagnosed malignancies from the nationwide network and registry of histopathology and cytopathology in the Netherlands (PALGA), which are confirmed and completed through the national hospital discharge databank. Data managers of the Netherlands Comprehensive Cancer Organization (IKNL) collect data on diagnosis, staging and treatment from medical records using international coding rules. The breast cancer stage is defined according to the TNM Classification of Malignant Tumors for breast cancer (6th edition) [13]. Vital status is available through linkage of NCR data with the Municipal Personal Records Database. Information on comorbidity and recurrence status was retrospectively collected from the medical records by trained data managers of the IKNL. All comorbidity, as present at the time of diagnosis, was recorded according to the categories in the ICD-10 classification, based on case record forms.

Patients diagnosed with stage I–III breast cancer aged 70 years or older diagnosed between 2003 and 2009, who underwent surgery, were included in this study. For patients diagnosed between 2003 and 2006, only patients from one of the nine Dutch registry regions were included, as information on comorbidity was at that time only available in this particular region. For patients diagnosed between 2007 and 2009, patients from all nine Dutch regions were included, as comorbidity and recurrence status were collected retrospectively specifically for this study. Patients with missing information on comorbidity and vital status were excluded. As death without distant recurrence was used as a proxy for other-cause mortality (described in next paragraph), patients with missing recurrence status were also excluded.

### 2.2. Definitions

The primary outcome was mortality from other causes than breast cancer, which was defined as death without distant recurrence, given that cause of death as registered on death certificates was not available. Another reason was that it is known that ascertaining the cause of death in older patients with breast cancer is prone to misclassification and tends to overattribute mortality to breast cancer [14]. As patients with early-stage breast cancer are unlikely to die from breast cancer without developing a distant recurrence, death without a distant recurrence was considered a valid proxy for other-cause mortality in prior research [8,15]. Moreover, no treatment-related mortality is present. No lethal postoperative complications are described, and no chemotherapy toxicity occurs as chemotherapy was discouraged for patients over 70 years in national guidelines at the time [16].

The specific comorbidities that were analyzed separately were comorbidities that are included in the Charlson comorbidity index or were present in at least 1% of the patients. Psychiatric diseases did not include dementia, which was reported separately. The Charlson comorbidity index was developed in 1987 to predict 1-year mortality in hospitalized patients (*n* = 604) and validated in patients with breast cancer [11,12]. Solid tumors, leukemia, lymphoma and AIDS were omitted because breast cancer was the index disease, and AIDS did not occur. The remaining 12 comorbidities had weights from 1 to 3. The sum of these weights is called the Charlson score. The Charlson index was compared with comorbidity count as this is the simplest comorbidity score. Given that other-cause mortality is our outcome of interest, all comorbidities with a potential impact on life expectancy were included in the comorbidity count. These comprised all comorbidities that required medication at the time of diagnosis or were judged to impact life expectancy based on clinical knowledge.

### 2.3. Statistical Analysis

Patients and treatment characteristics were described as frequencies and percentages. Comorbidity was described as frequencies and percentages of patients with specific comorbidities (yes; no), Charlson score (0; 1; 2; ≥3) and comorbidity count (0; 1; 2; ≥3). The distribution of the comorbidity scores was graphically presented. The relation between comorbidity and 5-year other-cause mortality was assessed by performing univariate and age-adjusted fine and gray analysis. Since the outcome of interest was other-cause mortality, distant recurrence was considered a competing event as a proxy for breast cancer deaths [17]. The associations are expressed as subdistribution hazard ratios (sHR) with 95% confidence intervals (CIs). For the specific comorbidities, patients without this comorbidity were used as reference. Charlson score 0 and zero comorbidities were used as a reference for the Charlson index and comorbidity count, respectively.

To compare the predictive value of the Charlson index and comorbidity count, first discrimination was assessed using c-statistics, which correspond to the area under the receiver operating characteristic (ROC) curve. The c-statistics of the univariable Charlson and comorbidity count fine and gray models were compared using the comorbidity scores as a continuous variable. To assess the additional value, improvements in c-statistics by adding the comorbidity scores to a model based on age alone were compared. A sensitivity analysis was performed to assess the potential effect of tumor characteristics on the relationship between comorbidity and other cause mortality by performing multivariate fine and gray models, including age, stage, grade and endocrine receptor status. The proportionality assumption was tested using Schoenfeld residuals. No violation of the assumption was found.

Next, calibration of the fine and gray models, including age and the comorbidity scores, was assessed by plotting the observed cumulative incidence of 5-year other-cause mortality against the predicted 5-year other-cause mortality. Using the cumulative incidence competing risk method, distant recurrence was considered a competing event as a proxy for breast cancer deaths. To make the calibration plots, patients were grouped in tenths according to the predicted cumulative incidences of 5-year other-cause mortality. The calibration plots were visually compared with the ideal x = y line.

Finally, as the c-statistic is substantially lower in the presence of competing events, an additional analysis was performed to evaluate the impact of comorbidity in addition to age [18]. For this reason, the cumulative incidence curves of other-cause mortality by comorbidity count were presented stratified by age (70–74 years; 75–79 years; 80 years and older). Stata SE 12.0 was used for the statistical analysis. All statistical tests were two-sided, and a *p*-value < 0.05 was considered statistically significant.

## 3. Results

**Figure 1:**
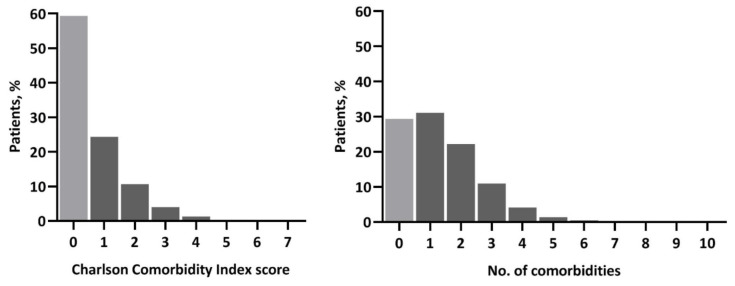
Distribution of comorbidity by measurement type.

Between 2003 and 2009, 19,748 patients aged 70 years or older were surgically treated for non-metastasized breast cancer, of which 1329 (6.7%) were excluded due to missing follow-up for recurrence or vital status. A total of 7511 patients with available information on comorbidity were included in the current study. The median age was 76.0 years (interquartile range 72.8–81.7 years). Patient and treatment characteristics are shown in Table 1. Most patients had stage I (43.9%) or stage II (43.4%) breast cancer. Of the 6382 patients with hormone receptor-positive disease, 56.2% received adjuvant endocrine treatment in line with the Dutch treatment guideline stating that patients with favorable tumor characteristics (grade 1 up to 2 cm and grade 2 up to 1 cm) do not receive adjuvant endocrine treatment as the absolute survival benefit is very limited in patients with a low-risk tumor. Only 2.6% of all patients received adjuvant chemotherapy. Figure 1 shows the distribution of the Charlson index and comorbidity count. In 29% of patients, zero comorbidities were counted, and 59% had a Charlson score of 0 caused by a considerable number of patients having comorbidities not included in the Charlson index. The prevalence of specific comorbidities is presented in Table 2. Of the 4460 patients with a Charlson score of 0, 2206 patients (49.5%) had one or more comorbidities on the count, particularly hypertension (Supplementary Table S1). After five years of follow-up, 1450 patients (19.3%) had died without a distant recurrence, 524 patients died after developing a distant recurrence (7.0%), and 135 were alive with a distant recurrence (1.8%).

### 3.1. Specific Comorbidities

**Figure 2:**
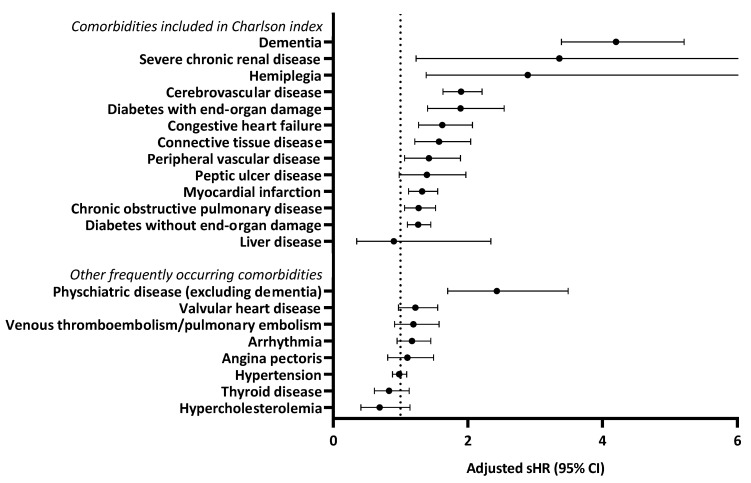
Adjusted subdistribution hazard ratios (sHRs) for 5-year other cause mortality by specific comorbidities. The multivariable model included all other specific comorbidities and age.

All individual comorbidities included in the Charlson index increased the risk of 5-year other-cause mortality in the univariate analysis except for liver disease (Supplementary Table S2). The age-adjusted sHRs are presented in Figure 2, with the sHR of peptic ulcer disease no longer significant after adjustment for age. The highest sHR was seen for dementia, which was associated with a fourfold higher risk of other-cause mortality compared to patients without dementia (age-adjusted sHR 4.22, 95% CI 3.41–5.23). Of the specific comorbidities not included in the Charlson index, the presence of arrhythmia, psychiatric disease (excluding dementia), and valvular heart disease increased the risk of other-cause mortality in univariate analysis (Supplementary Table S2). The sHRs for psychiatric disease remained significant after adjustment for age (Figure 2). Patients with a psychiatric disease had a more than two-fold increased risk of other-cause mortality compared with patients without the psychiatric disease (age-adjusted sHR 2.44, 95% CI 1.70–3.50).

### 3.2. Charlson Index

**Figure 3:**
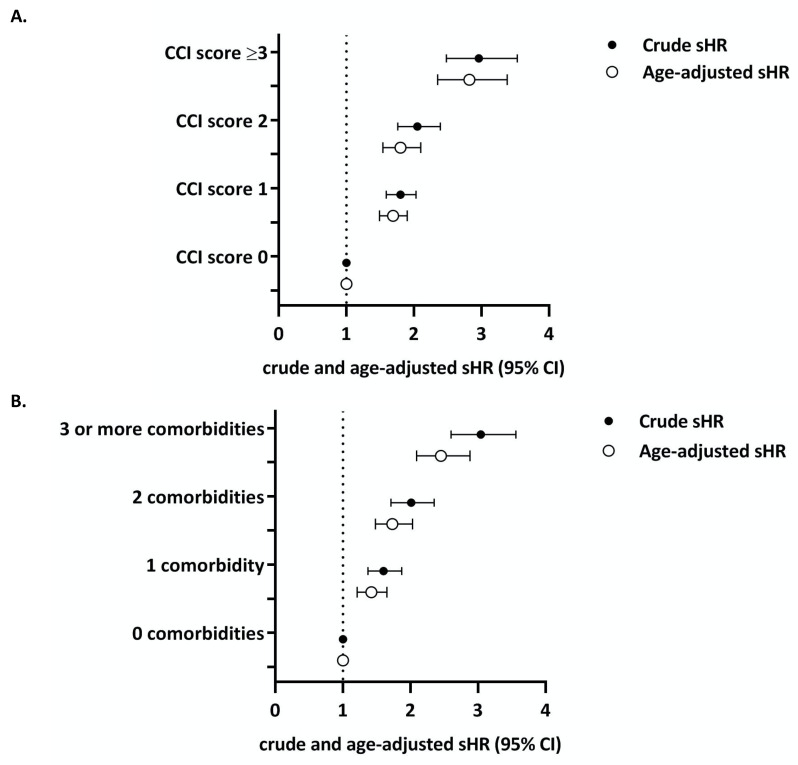
(**A**) Crude (●) and age-adjusted (○) subdistribution hazard ratios (sHRs) for 5-year other-cause mortality by Charlson index. (**B**) Crude (●) and age-adjusted (○) sHRs for 5-year other-cause mortality by comorbidity count.

Table 3 and Figure 3 show the crude and age-adjusted sHR for other-cause mortality by comorbidity score. With each increasing comorbidity category, patients had a higher risk of dying from other causes than patients with a Charlson score of 0 or zero comorbidity count, respectively. The sensitivity analysis showed no effect of tumor characteristics on the relationship between comorbidity and age on other cause mortality (Supplementary Table S3).

The c-statistic for predicting 5-year other-cause mortality was similar between the univariable models of the Charlson index (0.58, 95% CI 0.57–0.59) and comorbidity count (0.58, 95% CI 0.58–0.59). The c-statistic for predicting 5-year other-cause mortality based on age alone was 0.62 (95% CI 0.62–0.63), which increased to 0.65 (95% CI 0.64–0.66) by adding the Charlson index, and to 0.64 (95% CI 0.64–0.65) by adding comorbidity count (Table 3). Calibration was good for both the fine and gray models, including age and Charlson index and age and comorbidity count (Supplementary Figure S1).

**Figure 4:**
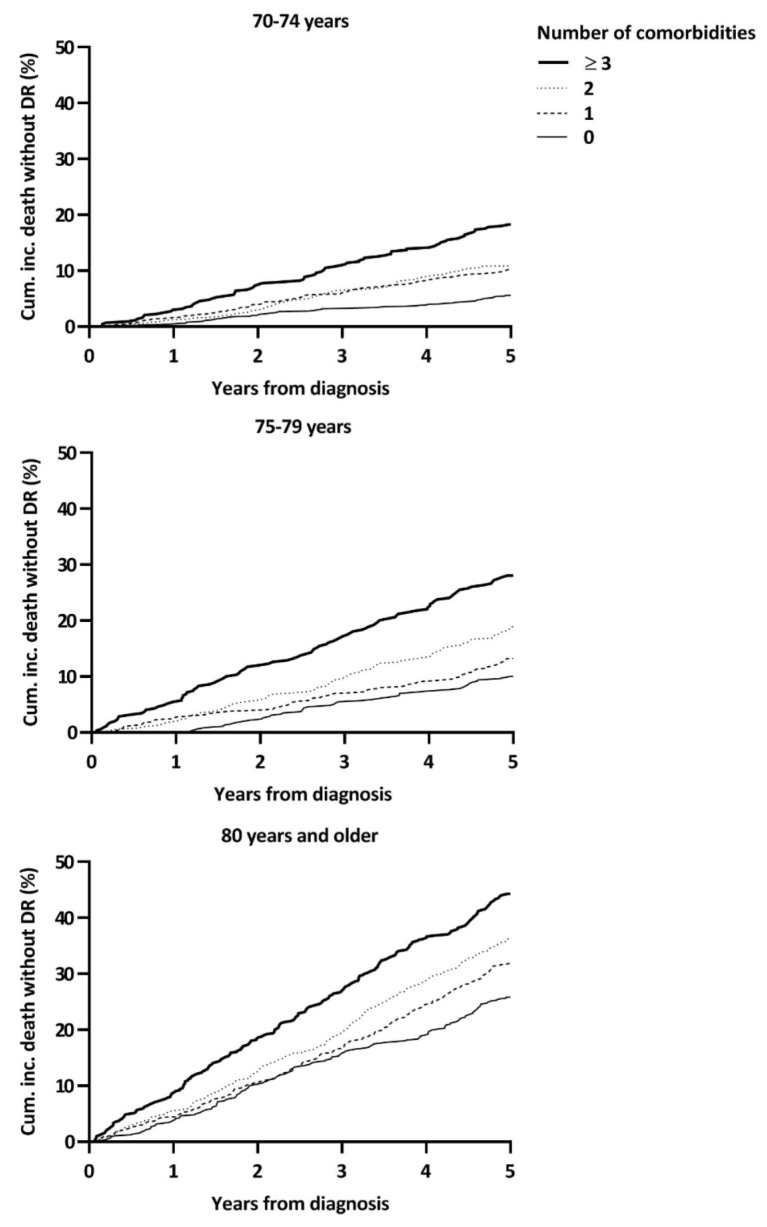
Cumulative incidences of other-cause mortality by age and number of comorbidities.

The impact of comorbidity in addition to age was also evaluated by stratifying the cumulative incidence curves of death from other causes by age and comorbidity count (Figure 4). These cumulative incidence curves demonstrated a clear trend between a higher comorbidity count and increasing other-cause mortality in all three age groups.

## 4. Discussion

The main finding of this study is that the predictive value of the Charlson index for 5-year other-cause mortality is similar to that of comorbidity count. Furthermore, of the specific comorbidities not included in the Charlson index, the only psychiatric disease was associated with an increased risk of other-cause mortality after adjustment for age.

It is well-known that comorbidity is associated with overall and other-cause mortality in patients with breast cancer. This was demonstrated in population-based [19,20,21,22,23,24,25] and trial-based [8,15] cohorts using the Charlson index [15,19,20,22,23,24] or comorbidity count [8,19,21]. Unlike the current study, a previous study found that prediction of other-cause mortality was better for comorbidity count than for the Charlson index [19]. However, in this previous study, while calculating deaths from other causes, breast cancer-specific deaths were censored rather than explicitly taken into account as a competing event [18]. Our study found that the Charlson index had a similar predictive value as the comorbidity count. Our data provide some clues that could explain this finding.

First, the weights could play a role. Although dementia gave a fourfold risk of dying from other causes in the present study, dementia is only assigned a weight of one in the Charlson index. Others have also suggested that the original Charlson weights may no longer be appropriate. A SEER-Medicare population-based cohort study of 64,034 patients with breast cancer aged 66 years or older demonstrated that dementia, congestive heart failure and COPD would be assigned a higher weight if Charlson’s method of assigning weights by rounding adjusted hazard ratio for overall mortality was applied [20]. Similarly, a Danish population-based cohort study of 59,673 postmenopausal patients with stage I-III breast cancer showed that dementia and COPD would be assigned a higher weight [26].

A second explanation could be that the Charlson index misses comorbidities that are relevant for the remaining life expectancy. This is suggested because 60% of the patients in our cohort of patients over 70 years had a Charlson score of 0, of which 35% had one comorbidity, and 16% had two or more comorbidities that are not included in the Charlson index. Similar rates of patients with a Charlson score of 0 were seen in the aforementioned population-based cohorts [20,22]. Psychiatric disease is not included in the Charlson index, but its presence was strongly associated with other-cause mortality in the present study. The association of psychiatric diseases with overall mortality also stood out in previous Dutch and American population-based studies [21,27]. Possibly, this is due to improved recognition and diagnosis of psychiatric diseases over the past years.

As can be expected, the strongest predictor for other-cause mortality is age. However, in line with others, our study demonstrated that comorbidity provides additional predictive value. First, the association with other-cause mortality remained after adjusting for age. Second, although modestly, the c-statistic improved by adding comorbidity to the model based on age alone. Third, cumulative incidence curves showed a clear trend between comorbidity and other-cause mortality stratified by age. Hence, the question is raised how comorbidity should be incorporated in prediction tools for clinical practice. As the Charlson index is the most widely known standardized comorbidity score, the present study evaluated the Charlson index for this purpose. Comorbidity count was used as a reference because this is the simplest comorbidity score as no checklist of specific comorbidities is needed. Based on our finding that the Charlson index performed similar to comorbidity count, we would argue against using the original Charlson index in the development of new prediction tools for older patients with breast cancer. Although changing the weights and adding new comorbidities, such as psychiatric diseases, could improve the predictive value of the original Charlson index, the implication that all the separate comorbidities would need to be included in the prediction tool reduces its practicality. In our opinion, the advantage of using comorbidity count is that its simplicity enhances the applicability of the tool in clinical practice. Future studies must clarify to what extent updated Charlson weights could improve its predictive value in comparison to comorbidity count.

Interestingly, the c-statistics of our models based on age and comorbidity score were lower compared to previous studies performed in similar study populations [10,19]. Several reasons could explain this. First, patients in the present study were somewhat older than previous studies, and the association between comorbidity and overall mortality seems to diminish with age [21]. Second, it is important to mention that the c-statistics in these previous studies are based on cox proportional hazards models, opposed to the competing risk models in the present study. This is relevant as the c-statistic is lower in the presence of competing events since patients with a high predicted risk of dying from other causes could still develop a distant recurrence [18]. It makes sense that if no such competing event can interfere with the prediction, the predictive accuracy will be better. Therefore, the predictive accuracy should not be based on the c-statistic alone, and the traditional interpretation may not be appropriate [18]. Since the age-adjusted sHRs and cumulative incidence curves stratified by age still showed a clear association between comorbidity and other-cause mortality, we believe that the modest improvement in c-statistic by adding comorbidity to a model based on age alone is a clinically relevant improvement.

Lastly, other geriatric parameters besides comorbidity status that discern life expectancy could improve prediction of other-cause mortality. For community-dwelling older individuals, it is known that prediction tools that include functional parameters obtained from a geriatric assessment can more accurately predict life expectancy [28]. For patients with breast cancer, the evidence also accumulates that using geriatric parameters in addition to traditional prognostic factors improves prediction [29,30,31]. Therefore, geriatric parameters should also be considered for new prediction tools. Our research group is currently working on such a tool in the *prediction of outcome, risk of toxicity and quality of life in older patients treated for breast cancer* (PORTRET) study. The aim is to incorporate tumor characteristics, such as tumor stage, grade and estrogen receptor status, comorbidity and other geriatric parameters to predict breast cancer and other-cause mortality, but also focus on other relevant outcomes, such as toxicity and functional outcomes.

A strength of this study was that it was performed in a large nationwide cohort with detailed information on comorbidity and follow-up. The population-based character enhances the generalizability of our results. Another strength was that we selected patients aged 70 years and older, as comorbidity influences treatment decisions in this age category. Last, fine and gray regression models that considered distant recurrence as competing events were used. The lack of information on the cause of death can be seen as a limitation, although ascertaining the cause of death in older patients with cancer is prone to misclassification [14]. Furthermore, as patients with early breast cancer are unlikely to die from breast cancer without developing a distant recurrence, using death without distant recurrence is a valid proxy for other-cause mortality also used by others [8,15]. It may be possible that we slightly underestimate other-cause mortality in *n* very small number of patients with limited recurrent disease (e.g., a solid bone metastasis), as these patients may be misclassified as having died due to breast cancer.

## 5. Conclusions

The Charlson index had no superior predictive value for other-cause mortality over comorbidity count in older patients with early breast cancer. To tailor a prediction tool to the older population with breast cancer, comorbidity status and other-cause mortality should be considered. To facilitate the application in clinical practice, we would argue the use of comorbidity count in new prediction tools. Future research is needed to assess the predictive value of other geriatric parameters for other-cause mortality, as these could further improve prediction.

## Figures and Tables

**Table 1:** Patient and treatment characteristics.

Patients	No. (%)
Total	7511
**Year of diagnosis**	
2003	309 (4.1)
2004	452 (6.0)
2005	548 (7.3)
2006	564 (7.5)
2007	1552 (20.7)
2008	1615 (21.5)
2009	2471 (32.9)
**Age category**	
70–74 years	3292 (43.8)
75–79 years	1778 (23.7)
≥80 years	2441 (32.5)
**TNM stage**	
I	3297 (43.9)
II	3259 (43.4)
III	944 (12.6)
Unknown	11 (0.2)
**Tumor grade**	
1	1847 (24.6)
2	3387 (45.1)
3	1803 (24.0)
Unknown	474 (6.3)
**Hormone receptor status**	
ER- and/or PR-positive	6382 (85.0)
ER- and PR-negative	968 (12.7)
Unknown	180 (2.3)
**Her2 status**	
Positive	610 (8.0)
Negative	5667 (75.1)
Unknown	1269 (16.8)
**Type of surgery**	
Mastectomy	4346 (57.9)
Breast-conserving surgery	3165 (42.1)
Endocrine treatment *	
Yes	3584 (56.2)
No	2798 (43.8)
**Chemotherapy**	
Yes	194 (2.6)
No	7317 (97.4)

* percentage of patients with hormone receptor-positive breast cancer.

**Table 2:** Prevalence of specific comorbidities.

Comorbidity	No. (%)
**Comorbidities included in Charlson index**	
Myocardial infarction	671 (8.9)
Congestive heart failure	216 (2.9)
Peripheral vascular disease	216 (2.9)
Cerebrovascular disease	545 (7.3)
Dementia	164 (2.2)
Chronic obstructive pulmonary disease	620 (8.3)
Connective tissue disease	212 (2.8)
Peptic ulcer disease	128 (1.7)
Liver disease	31 (0.4)
Diabetes without end-organ damage	1219 (16.2)
Diabetes with end-organ damage	162 (2.2)
Hemiplegia	16 (0.2)
Severe chronic renal disease	12 (0.2)
Other frequently occurring comorbidities *	
Hypertension	2971 (39.6)
Arrhythmia	342 (4.6)
Valvular heart disease	294 (3.9)
Thyroid disease	293 (3.9)
Venous thromboembolism/pulmonary embolism	213 (2.8)
Angina pectoris	166 (2.2)
Tuberculosis	100 (1.3)
Hypercholesterolemia	94 (1.3)
Psychiatric disease (excluding dementia)	90 (1.2)

* present in ≥1% of the study cohort.

**Table 3:** Crude and age-adjusted subdistribution hazard ratios for 5-year other-cause mortality by Charlson index and comorbidity count and corresponding model c-statistics.

Comorbidity Category	Charlson Index		Comorbidity Count	
	Crude sHR (95% CI)	Age-Adjusted sHR (95% CI)	Crude sHR (95% CI)	Age-Adjusted sHR (95% CI)
0	Referent	Referent	Referent	Referent
1	1.80 (1.59 to 2.03)	1.69 (1.49 to 1.90)	1.60 (1.37 to 1.87)	1.42 (1.21 to 1.65)
2	2.05 (1.76 to 2.39)	1.80 (1.54 to 2.10)	2.01 (1.71 to 2.35)	1.73 (1.48 to 2.03)
≥3	2.96 (2.49 to 3.53)	2.82 (2.35 to 3.38)	3.04 (2.60 to 3.56)	2.45 (2.09 to 2.88)
Model c-statistic (95% CI) *	0.58 (0.57 to 0.59)	0.65 (0.64 to 0.66)	0.58 (0.58 to 0.59)	0.64 (0.64 to 0.65)

* c-statistics of the age-adjusted models correspond to the models, including age and the comorbidity score.

## Data Availability

The datasets generated during and analyzed during the current study are available from the corresponding author on reasonable request.

## Supplementary Materials

The following are available online at https://www.mdpi.com/article/10.3390/cancers13071627/s1, Figure S1: Calibration of the two fine and gray models for prediction of 5-year other-cause mortality, including age and Charlson score (blue), and age and comorbidity count (red), Table S1: Comorbidity count and specific comorbidities in patients with a Charlson comorbidity index score 0 and ≥1; Table S2: Subdistribution hazard ratios (sHRs) with 95% confidence intervals of specific comorbidities for 5-year other-cause mortality. Patients without the specific comorbidity were used as referents. Table S3: Sensitivity analysis. Crude and multivariate subdistribution hazard ratios for 5-year other-cause mortality by Charlson index and comorbidity count. The first multivariate model was adjusted for age alone, and the second multivariate model was adjusted for age and tumor characteristics: stage, grade and endocrine receptor status.
