# Supplementary material for: Prediction of Other-Cause Mortality in Older Patients with Breast Cancer Using Comorbidity
Source: Cancers (Basel). 2021 Apr 1;13(7):1627. doi: 10.3390/cancers13071627 (PMC8036543; doi:10.3390/cancers13071627)
Supplement: Supplementary file 1 [file cancers-13-01627-s001.pdf]

cancers

MDPI

Article

Prediction of Other-Cause Mortality in Older Patients with Breast Cancer Using Comorbidity

Anna Z. de Boer [1,2], Esther Bastiaannet [1,2], Hein Putter [3], Perla J. Marang-van de Mheen [4], Sabine Siesling [5,6], Linda de Munck [5], Kelly M. de Ligt [5,6], Johanneke E.A. Portielje [2], Gerrit Jan Liefers [1] and Nienke A. de Glas [2,*]

[1] Department of Surgery, Leiden University Medical Center, 2333 ZA Leiden, The Netherlands; a.z.de_boer@lumc.nl (A.Z.d.B.); e.bastiaannet@lumc.nl (E.B.); g.j.liefers@lumc.nl (G.J.L.)
[2] Department of Medical Oncology, Leiden University Medical Center, 2333 ZA Leiden, The Netherlands; j.e.a.portielje@lumc.nl
[3] Department of Medical Statistics, Leiden University Medical Center, 2333 ZA Leiden, The Netherlands; h.putter@lumc.nl
[4] Department of Medical Decision Making, Leiden University Medical Center, 2333 ZA Leiden, The Netherlands; p.j.marang-van_de_mheen@lumc.nl
[5] Department of Research and Development, Netherlands Comprehensive Cancer Organization, 3500 BN Utrecht, The Netherlands; s.siesling@iknl.nl (S.S.); l.demunck@iknl.nl (L.d.M.); k.d.ligt@nki.nl (K.M.d.L.)
[6] Department of Health Technology and Services Research, Technical Medical Center, University of Twente, 7522 NB Enschede, The Netherlands
* Correspondence: n.a.de_glas@lumc.nl

Supplementary

Citation: de Boer, A.Z.; Bastiaannet, E.; Putter, H.; Marang-van de Mheen, P.J.; Siesling, S.; de Munck, L.; de Ligt, K.M.; Portielje, J.E.A.; Liefers, G.J.; de Glas, N.A. Prediction of Other-Cause Mortality in Older Patients with Breast Cancer Using Comorbidity. Cancers 2021, 13, 1627. https://doi.org/10.3390/cancers13071627

Academic Editor: David Wong

Received: 1 February 2021
Accepted: 25 March 2021
Published: 1 April 2021

Publisher's Note: MDPI stays neutral with regard to jurisdictional claims in published maps and institutional affil-iations.

Copyright: © 2021 by the authors. Licensee MDPI, Basel, Switzerland. This article is an open access article distributed under the terms and conditions of the Creative Commons Attribution (CC BY) license (http://creativecommons.org/licenses/by/4.0/).

Table S1. Comorbidity count and specific comorbidities in patients with a Charlson Comorbidity Index score 0 and ≥1.

	Charlson Score 0	Charlson Score ≥1
	No. (%)	No. (%)
Comorbidity count		
0	2206 (49.5)	0 (0)
1	1556 (34.9)	782 (25.63)
2	536 (12)	1134 (37.17)
≥3	162 (3.6)	1135 (37.2)
Comorbidities not included in the CCI		
Hypertension	1503 (33.7)	1468 (48.1)
Arrhythmia	176 (4)	166 (5.4)
Valvular heart disease	157 (3.5)	137 (4.5)
Thyroid disease	169 (3.8)	124 (4.1)
Venous thromboembolism/pulmonary embolism	102 (2.3)	111 (3.6)
Angina pectoris	57 (1.3)	109 (3.6)
Hypercholesterolemia	49 (1.1)	45 (1.5)
Psychiatric disease (excluding dementia)	57 (1.3)	33 (1.1)

Table S2. Subdistribution hazard ratios (sHRs) with 95% confidence intervals of specific comorbidities for 5-year other-cause mortality. Patients without the specific comorbidity were used as referent.

	No. (%)	Crude sHR (95% CI)	Adjusted sHR* (95% CI)
Specific comorbidity included in CCI			
Myocardial infarction	671 (8.9)	1.57 (1.34 to 1.83)	1.32 (1.12 to 1.55)
Congestive heart failure	216 (2.9)	2.67 (2.15 to 3.31)	1.62 (1.27 to 2.07)
Peripheral vascular disease	216 (2.9)	1.45 (1.10 to 1.90)	1.42 (1.06 to 1.90)
Cerebrovascular disease	545 (7.3)	2.54 (2.19 to 2.93)	1.90 (1.63 to 2.21)
Dementia	164 (2.2)	5.95 (4.89 to 7.23)	4.22 (3.41 to 5.23)
Chronic obstructive pulmonary disease	620 (8.3)	1.38 (1.17 to 1.63)	1.26 (1.05 to 1.51)
Connective tissue disease	212 (2.8)	1.56 (1.21 to 2.01)	1.56 (1.21 to 2.03)
Peptic ulcer disease	128 (1.7)	1.63 (1.17 to 2.27)	1.38 (0.97 to 1.97)
Liver disease	31 (0.4)	0.85 (0.34 to 2.10)	0.90 (0.35 to 2.33)
Diabetes without end to organ damage	1219 (16.2)	1.30 (1.14 to 1.48)	1.26 (1.10 to 1.45)
Diabetes with end to organ damage	162 (2.2)	1.61 (1.22 to 2.14)	1.90 (1.41 to 2.55)
Hemiplegia	16 (0.2)	4.18 (2.07 to 8.45)	2.91 (1.38 to 6.10)
Severe chronic renal disease	12 (0.2)	4.74 (2.02 to 11.09)	3.38 (1.24 to 9.25)
Other frequently occurring comorbidities			
Hypertension	2971 (39.6)	1.07 (0.96 to 1.19)	0.98 (0.88 to 1.09)
Arrhythmia	342 (4.6)	1.67 (1.36 to 2.06)	1.18 (0.95 to 1.45)
Valvular heart disease	294 (3.9)	1.45 (1.15 to 1.83)	1.23 (0.97 to 1.55)
Thyroid disease	293 (3.9)	0.93 (0.70 to 1.24)	0.83 (0.61 to 1.13)
Venous thrombo-/pulmonary embolism	213 (2.8)	1.29 (0.98 to 1.71)	1.20 (0.91 to 1.58)
Angina pectoris	166 (2.2)	1.31 (0.97 to 1.78)	1.10 (0.81 to 1.49)
Hypercholesterolemia	94 (1.3)	0.73 (0.44 to 1.23)	0.69 (0.41 to 1.14)
Psychiatric diseases (excluding dementia)	90 (1.2)	1.64 (1.13 to 2.37)	2.44 (1.70 to 3.50)

*The multivariable model included all specific comorbidities and age.

Table S3. Sensitivity analysis. Crude and multivariate subdistribution hazard ratios for 5-year other-cause mortality by Charlson index and comorbidity count. The first multivariate model was adjusted for age alone, and the second multivariate model was adjusted for age and tumor characteristics: stage, grade and endocrine receptor status.

	Charlson Index			Comorbidity Count		
Comorbidity Category	Crude	Age-Adjusted	Adjusted for Age and Tumor Characteristics*	Crude	Age-Adjusted	Adjusted for Age and Tumor Characteristics*
	sHR (95% CI)	sHR (95% CI)	sHR (95% CI)	sHR (95% CI)	sHR (95% CI)	sHR (95% CI)
0	Referent	Referent	Referent	Referent	Referent	Referent
1	1.80 (1.59 to 2.03)	1.69 (1.49 to 1.90)	1.71 (1.50 to 1.94)	1.60 (1.37 to 1.87)	1.42 (1.21 to 1.65)	1.45 (1.24 to 1.71)
2	2.05 (1.76 to 2.39)	1.80 (1.54 to 2.10)	1.76 (1.50 to 2.07)	2.01 (1.71 to 2.35)	1.73 (1.48 to 2.03)	1.71 (1.45 to 2.01)
≥3	2.96 (2.49 to 3.53)	2.82 (2.35 to 3.38)	2.89 (2.38 to 3.50)	3.04 (2.60 to 3.56)	2.45 (2.09 to 2.88)	2.40 (2.03 to 2.84)

*Multivariate model including age, stage, grade and hormone receptor status.

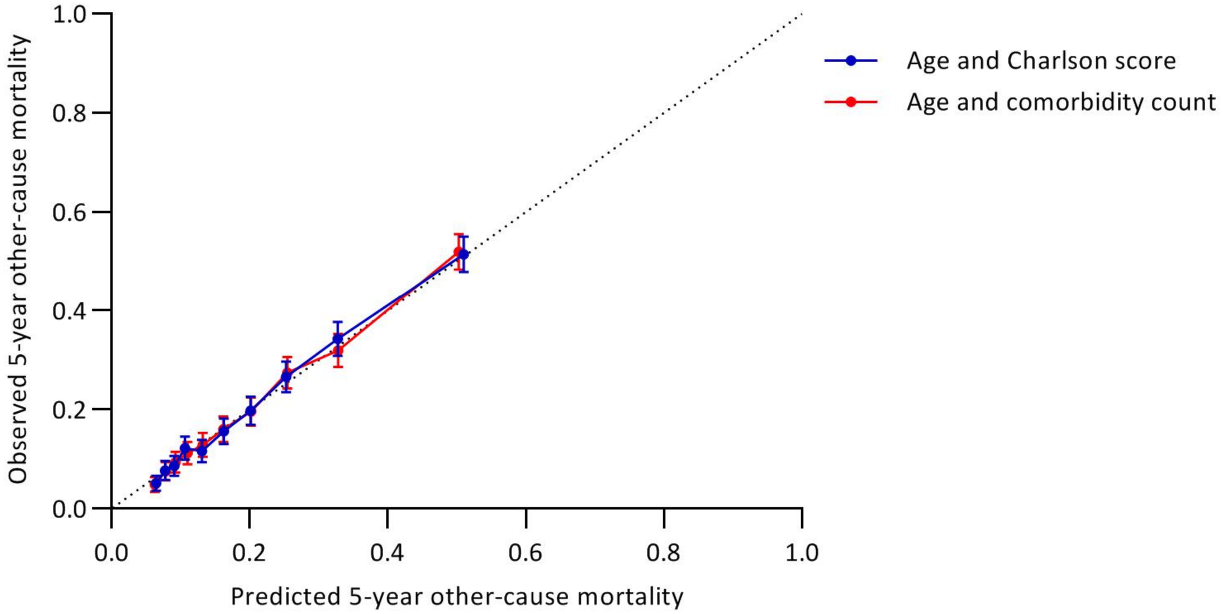

Figure S1. Calibration of the two Fine and Gray models for prediction of 5-year other-cause mortality, including age and Charlson score (blue), and age and comorbidity count (red).
